# Success Rate of Endotracheal Intubation Using Inline Stabilization with and without Cervical Hard Collar; a Comparative Study

**Published:** 2020-10-10

**Authors:** Welawat Tienpratarn, Chaiyaporn Yuksen, Kasamon Aramvanitch, Karn Suttapanit, Yahya Mankong, Nussareen Yaemluksanalert, Sansanee Meesawad

**Affiliations:** 1 Department of Emergency Medicine, Faculty of Medicine, Ramathibodi Hospital, Mahidol University, Bangkok 10400, Thailand.

**Keywords:** Intubation, Intratracheal, Spinal Injuries, Multiple trauma, Restraint, Physical

## Abstract

**Introduction:**

Application of a rigid cervical collar may interfere with the laryngeal view, and potentially lead to failed endotracheal intubation (ETI). This study aimed to compare intubation success rates while performing inline stabilization with and without cervical hard collar.

**Methods:**

This randomized prospective comparative study included paramedics working in the Department of Emergency Medicine, Ramathibodi Hospital, Mahidol University, Bangkok, Thailand to compare the success rates of endotracheal intubation on manikin using inline stabilization with and without cervical hard collar.

**Results:**

125 participants were evaluated; 63 in the rigid cervical collar and 62 in the non-cervical hard collar group. The rate of successful intubation was significantly higher using manual stabilization without cervical hard collar (61 (96.8%) vs. 55 (88.7%); p=0.048). The time required to successfully perform intubation was also shorter, with manual stabilization only (14.1 ±20.9 vs. 18.9±29.0; p = 0.081).

**Conclusion:**

It seems that, removal of the rigid cervical collar during ETI in patients with suspected traumatic spine injury could increase the intubation success rate.

## Introduction

Traumatic Spinal Injury (TSI) is one of the most common life-threatening traumatic conditions. It can injure nearby organs, such as the spinal cord, which can cause severe complications ranging from minor injuries to disability or death (1). There is an average incidence of 10.5 spinal injuries per 100,000 people worldwide or approximately 768,473 incidents per year. The most common cause is traffic accidents followed by falls; about 48.8% of spinal injuries require surgery (2).

In patients with suspected TSI, transportation requires a rigid cervical collar, head immobilization device, and spinal support throughout the lifting and moving procedure (3). In patients with a threatened airway, essential preliminary treatments are required for preventing airway obstruction and providing adequate ventilation support (1). In TSI patients, tracheal intubation must be performed carefully to prevent further injury to the cervical spine and spinal cord (4). Moreover, manual inline stabilization using a rigid cervical collar is recommended to reduce the movement of the cervical spine during tracheal intubation (5).

Using a rigid cervical collar and performing manual inline stabilization in patients suspected to have a cervical TSI can reduce the intubation-related movement of the cervical spine and aggravation of TSI (6). But the application of a rigid cervical collar can cause difficulty in airway management, decrease the inter-incisor, increase the Mallampati classification, decrease the angles of neck extension, and reduce cervical spine movements (7). Removing the rigid cervical collar or anterior portion during tracheal intubation has been suggested by an expert physician. 

Manual inline stabilization is a maneuver in which the care provider is standing at the patient's head, using the palms of both hands to grip the sides of the patients' head, which is reportedly a safe technique to perform during intubation. Using a rigid cervical collar and performing manual inline stabilization in patients suspected to have a cervical TSI can reduce the intubation-related movement of the cervical spine (8).

However, no reports have clearly described intubation success rates while using a rigid cervical collar and whether it is necessary to remove the rigid cervical collar during intubation. This study aimed to compare intubation success while using a rigid cervical collar along with manual inline stabilization versus manual inline stabilization alone when performed by paramedics on a manikin.

## Methods

***Study design and setting***

This randomized prospective comparative study included paramedics working at the Department of Emergency Medicine, Ramathibodi Hospital, Mahidol University, Bangkok, Thailand to compare the success rate of endotracheal intubation on manikin using inline stabilization with and without cervical hard collar. This study was approved by the institutional Ethics Committee Board of Ramathibodi Hospital Faculty of Medicine, Mahidol University. The Ethics Approval Reference Number is MURA2018/900. 

***Participants***

**Figure 1:**
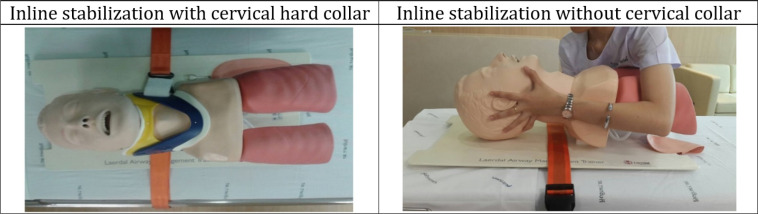
Endotracheal intubation using inline stabilization with and without rigid cervical collar on manikin

Eligible paramedic students who provided a signed consent to participate in the research were included and those who refused to participate were excluded. Participants were randomly assigned to endotracheal intubation using inline stabilization either with rigid cervical collar or without it using sequentially numbered opaque sealed envelopes and six-block randomization. Participants in the rigid cervical collar group performed intubation with a rigid cervical collar and manual inline stabilization and others performed intubation using manual inline stabilization without a rigid cervical collar (figure 1). 

**Definitions and procedures**

The data recorded in this study included the success of intubation within 60 seconds. Successful intubation was defined as the endotracheal tube's insertion into the manikin's bronchus in a maximum of two attempts. The successful intubation time was defined as the period from passing the equipment through the axial plane of manikin's front teeth to successful intubation. The viewpoint of intubation was defined as the viewpoint of the larynx during intubation, based on Mallampati classification.

The manikin was not moved, nor was the rigid collar removed. The rigid cervical collar position was measured each time intubation was conducted to ensure it was in the same position. Only one set of equipment, comprising of a Macintosh laryngoscope and No. 3 blades, was used in the study. Only one No. 7 endotracheal tube was used in the study. The equipment was tested for readiness before each intubation procedure, and the manikin was fixed in the original location and position. The duration of the study was two months, and we used the same manikin and equipment. 

**Data gathering**

The researcher recorded demographic variables of participants, intubation success, time to the success of intubation, and the laryngeal view grade for each participant, using a predesigned checklist.

**Statistical analysis**

For sample size estimation, we used the data from a pilot study of 20 paramedics intubating a manikin. The pilot study was done in Ramathibodi Hospital. The rate of intubation success within 60s in groups with and without collar was 92% and 70%, respectively. With a 95% confidence interval and power of 80%, the sample size ratio 1:1, and p = 0.05, the two‑sided test found that the minimum sample size should be 58 subjects. 

Chi‑square and Fisher’s exact tests were used to compare the categorical data. McNemar’s test and t‑test of rank‑sum tests were used for the comparison of continuous data. All statistical analyses were performed using Stata software version 14 (StataCorp, College Station, TX, USA). P < 0.05 was considered statistically significant.

## Results

125 participants were evaluated; 63 in the rigid cervical collar and 62 in the non-cervical hard collar group. Table 1 compares the baseline characteristics of participants. There were no statistically significant differences between groups regarding age (p =0.925), sex (p = 0.864), weight (p = 0.889), height (p = 0.985), and intubation experience (p = 0.878). The success of intubation was significantly higher with only manual inline stabilization compared to using the rigid cervical collar and manual inline stabilization (61 (96.8%) vs. 55 (88.7%); p = 0.048). The intubation time was shorter with only manual inline stabilization (14.1 ±20.9 vs. 18.9±29.0; p = 0.081). Comparison between the intubation characteristics of the 2 groups is presented in table 2. 

## Discussion

The results showed that the overall rate of successful intubation was significantly higher when using manual inline stabilization alone. Additionally, the laryngeal view grade was not significantly different between the two groups. 

In every trauma patient with suspected TSI, the cervical spine should be manually maintained in a neutral position before the primary survey and resuscitation. Applying a rigid cervical collar, stabilizing the body to a spinal board, and adequate external immobilization of the head should be performed. En route to the trauma center or emergency department (ED), if the TSI patients need assisted ventilation or endotracheal tube intubation, cervical collar might better be removed. On the scene or en route, ETI should be successful in a short period to prevent hypoxia and aspiration. It seems beneficial to remove the rigid cervical collar during ETI because it increases the success rate and shortens intubation time.

On scene or en route ETI is more difficult than ED intubation and a video laryngoscope, which improves laryngeal views, may not be available in prehospital care. Emergency medical service providers could remove the cervical collar and only apply manual inline stabilization before ETI for improving laryngeal views.

One study that evaluated intubation of patients using the rigid cervical collar and manual inline stabilization showed that both methods could stabilize the cervical spine. However, using rigid cervical collar, intubation success rate was reduced, and the laryngeal view was impaired. In addition, the applied methods were shown to significantly increase the time of intubation (9).

Another study found that using a rigid cervical collar could reduce the movement of the spine, but performing ETI was significantly more difficult (7).

The study by Kleine-Brueggeney M. et al. shows that the rigid cervical collar can cause limitations in mouth opening and significantly increase intubation time (10). The study by Yuk M. et al. was performed on 76 healthy volunteers in a simulation study and showed that the LEMON criteria (mouth opening, modified Mallampati classification, and neck extension) worsened significantly after rigid cervical collar application (7). 

 The study by Yuksen C, comparing the efficiency of intubation between a video laryngoscope and Macintosh laryngoscope on a manikin with a hard collar that restricted neck movement, showed that intubation success was significantly lower when using the Macintosh laryngoscope compared to when a video laryngoscope was used in the group of personnel who had experience performing intubation. In the inexperienced group, however, the time to successful intubation was not different between the two methods (11).

This study was done including senior paramedic students whose endotracheal intubation skill was similar to paramedic physicians. The results of this study may be used in the practice of ETI to remove the rigid cervical collar, especially in a prehospital setting with time limitation.

**Table 1:** Comparing the baseline characteristics of participants between groups

**Variables**	**Intubation groups**		**P values**
	**Inline (n = 63)**	**Inline + Collar (n = 62)**	
**Age (year)**			
Mean ± SD	23.5±5.1	23.2±4.2	0.925
**Gender n (%)**			
Male	32 (50.8)	31 (50.0)	0.864
Female	31(49.2)	31 (50.0)	
**Weight** ** (kg)**			
Mean ± SD	63.9 ± 16.1	64.5 ± 9.5	0.889
**Height** ** (m)**			
Mean ± SD	166.7 ± 8.0	166.4 ± 18.7	0.985
**Intubation experience** ** (year)**			
Pre-clinical year paramedics	45 (71.4)	44 (71.0)	0.878
Clinical year paramedics	18 (28.6)	18 (29.0)	

Data are presented as mean ± standard deviation (SD) or number (%).

**Table 2:** Comparing the intubation characteristics between groups

**Variables**	**Intubation groups**		** p**
	**Inline (n = 63)**	**Inline + Collar (n = 62)**	
**Successful intubation **			
Number (%)	61 (96.8)	55 (88.7)	0.048
**Time to successful intubation (second)**			
Mean ± SD	14.1 ±20.9	18.9±29.0	0.081
**Attempts of successful intubations N (%)**			
1	52 (82.5)	50 (80.6)	0.098
2	9 (14.3)	5 (8.1)	
≥3 (failed)	2 (3.2)	7 (11.3)	
Laryngeal view grade* N (%)			
1	19 (30.1)	14 (22.6)	0.675
2	32 (50.8)	32 (51.6)	
3	11 (17.5)	15 (24.2)	
4	1 (1.6)	1 (1.6)	
**Time to first successful intubation (second)**			
Mean ± SD	17.4 ± 14.4	18.8 ± 11.7	0.592

*Based on Mallampati classification.

## Limitations

There are some limitations to this study. First, the results of this study may not apply to real clinical situations as they were found using a manikin; but they may be used as preliminary data for further research. Second, using a manikin might have caused bias in evaluating the mallampati score, which should be further investigated by assessing the participant's laryngeal view report. Deterioration of the equipment such as an endotracheal tube, guidewire, and energy of battery of Macintosh laryngoscope might have caused bias and confounded our study results. 

## Conclusion:

It seems that, removal of the rigid cervical collar during ETI in patients with suspected TSI could increase the intubation success rate. 

## Data Availability

The data are not available for public access because of participants privacy concern but are available from the corresponding author upon reasonable request.
